# Adsorption of Cd (II) on Modified Granular Activated Carbons: Isotherm and Column Study

**DOI:** 10.3390/molecules22122280

**Published:** 2017-12-20

**Authors:** Paola Rodríguez-Estupiñán, Alessandro Erto, Liliana Giraldo, Juan Carlos Moreno-Piraján

**Affiliations:** 1 Faculty of Sciences, Department of Chemistry, Research Group on Porous Solids and Calorimetry, Universidad de Los Andes, Carrera 1ra # 18A-12, Bogotá 111711, Colombia; jp.rodrigueze@uninades.edu.co; 2 Department of Chemical Engineering, Materials and Industrial Production, Università degli Studi di Napoli Federico II, Piazzale Tecchio 80, 80125 Napoli, Italy; aleserto@unina.it; 3 Faculty of Sciences, Department of Chemistry, Universidad Nacional de Colombia, Carrera 30 # 45-03, Bogotá 111321, Colombia; lgiraldogu@unal.edu.co

**Keywords:** modified activated carbon, adsorption, cadmium, fixed bed column, oxidation treatment, kinetic model

## Abstract

In this work, equilibrium and dynamic adsorption tests of cadmium Cd (II) on activated carbons derived from different oxidation treatments (with either HNO_3_, H_2_O_2_, or NaOCl, corresponding to GACoxN, GACoxP, and GACoxCl samples) are presented. The oxidation treatments determined an increase in the surface functional groups (mainly the acidic ones) and a decrease in the pH_PZC_ (except for the GACoxCl sample). A slight alteration of the textural parameters was also observed, which was more significant for the GACoxCl sample, in terms of a decrease of both Brunauer-Emmett-Teller (*BET*) surface area and micropore volume. Adsorption isotherms were determined for all the adsorbents and a significant increase in the adsorption performances of the oxidized samples with respect to the parent material was observed. The performances ranking was GACoxCl > GACoxP > GACoxN > GAC, likely due to the chemical surface properties of the adsorbents. Dynamic tests in a fixed bed column were carried out in terms of breakthrough curves at constant Cd inlet concentration and flow rate. GACoxCl and GACoxN showed a significantly higher value of the breakpoint time, likely due to the higher adsorption capacity. Finally, the dynamic tests were analyzed in light of a kinetic model. In the adopted experimental conditions, the results showed that mass transfer is controlled by internal pore diffusion, in which surface diffusion plays a major role.

## 1. Introduction

Heavy metals are introduced to the environment through both natural sources, such as volcanic activity and leaching or erosion of soils (minerals) and anthropogenic sources, from different industrial processes such as mining, tannery, oil refining, metal coverings, and production of batteries, pesticides, and fertilizers [1,2,3]. Among heavy metals, cadmium (Cd) is considered as one of the most dangerous pollutants [4]. Recent studies have shown a direct correlation between exposure to cadmium and different adverse effects over health, like renal and gastrointestinal damage [5], irritation to respiratory tissue, hematological effects [6,7], effects over reproductive system [8,9], endocrine disorder [10], etc. Moreover, cadmium is considered as a potentially carcinogenic (particularly for lung and prostate cancer) [11] and genotoxic agent [12,13], and is involved in musculoskeletal effects like Osteomalacia, or ‘itai-itai’ disease [14,15,16].

According to the Agency for Toxic Substances and Disease Registry (ATSDR), the demand for cadmium in the nickel-cadmium (Ni-Cd) battery industry is increasing [17], while it is decreasing in other application areas, like coatings and pigments, due to environmental concerns and regulations [4,18]. In some cases, primary production of cadmium may decrease as zinc prices increase, since producers may choose to discard the cadmium byproduct instead of refining it. Consequently, water contamination by heavy metal is still a pressing environmental problem that is urgent to solve [19,20].

Several technologies have been proposed for the removal of heavy metals, including cadmium, from polluted waters, which include chemical precipitation, chemical electrodeposition, electro-dialysis [21], coagulation-flocculation [22,23], ionic exchange [24], reverse osmosis [25], ultrafiltration [26], and adsorption [27,28]. Among these techniques, adsorption has become a simple, environmentally friendly, and economically viable technique. In this field, activated carbon has been extensively used as adsorbent for the capture of organic and inorganic pollutants from aqueous media [29]; however, its general low selectivity has driven to the study of ad hoc chemical modifications of its surface, with the objective to obtain a greater affinity towards specific ions in solution [19,27].

On the other hand, adsorption isotherms are invaluable tools that provide a measure of the maximum adsorption capacity as well as the best operation conditions of the adsorption system (e.g., concentration, pH and ionic strength of the solution, temperature, etc.) [30,31,32,33,34]. On the contrary, dynamic adsorption studies (e.g., in a fixed bed column) provide complementary information, such as mass transfer rate, kinetic regimes, length of unused bed at breakpoint, etc., as the operating conditions are not under equilibrium because a feed solution is continuously fed into the column, and a non-stationary mass transfer process is established between a mobile phase (water) and the solid phase (adsorbent). Industrial processes are generally carried out under continuous conditions; therefore, this type of studies provides the most real operative conditions for wastewater treatment [35,36,37,38,39].

The main purpose of this study was to elucidate some of the factors determining the selectivity and efficiency toward Cd adsorption of four different activated carbons obtained starting from a same precursor (GAC), which was subjected to different oxidation treatments with either nitric acid (GACoxN), hydrogen peroxide (GACoxP), or sodium hypochlorite (GACoxCl) solutions. All the adsorbents were fully characterized both chemically and texturally by different techniques, i.e., N_2_ and CO_2_ physisorption at −196 °C and 0 °C, respectively, Boehm titration method, and SEM analysis. Thermodynamic adsorption tests were carried out at constant temperature (20 °C) in order to correlate the properties of the solids with the retrieved Cd (II) adsorption capacity, and to support the subsequent dynamic tests. Fixed-bed tests were carried out at the same temperature in order to assess the kinetic properties of the system and to define the best performing adsorbent in simulating real operating conditions. Finally, the analysis of both thermodynamic and kinetic aspects of the adsorption systems was corroborated by dedicated modelling analyses. 

## 2. Results and Discussion

### 2.1. SEM Analysis

**Figure 1:**
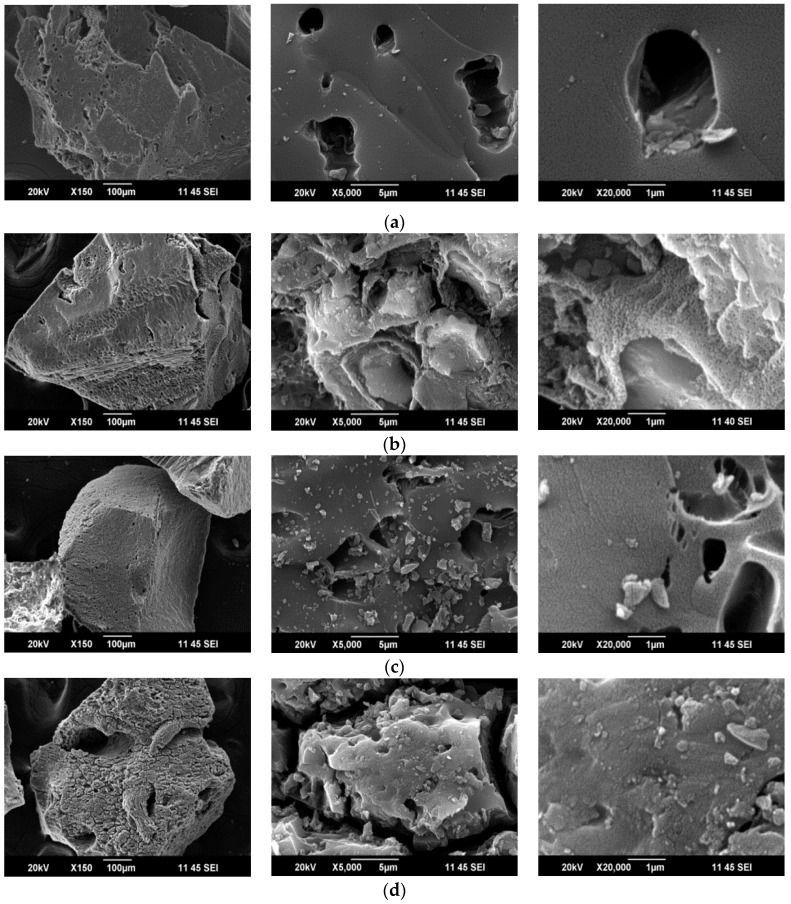
SEM micrograph at different magnifications for (**a**) GAC; (**b**) GACoxN; (**c**) GACoxP; and (**d**) GACoxCl.

Scanning electron microscopy (SEM) provides useful information about the morphology and topology of the solid surfaces. The micrographs obtained by SEM are presented in Figure 1a–d, in which the surface of the activated carbons and their modifications can be observed.

In the micrographs of the GAC sample (Figure 1a), it is possible to observe the morphology of the outer surface, which has cavities with a high degree of roughness; observed at micrometer scale. These spaces are of the order of 1 μm [40]. Although the micropores and mesopores are not visible, the micrographs present the forms and location of the macropores on the surface of the solid. The macropores favor the diffusion processes and are formed during the activation by the effect of the oxidation gas, in this case the CO_2_, and the loss of volatile material during the pyrolysis [40,41].

The oxidation with nitric acid (Figure 1b) has a visual impact on the surface morphology, with a loss in uniformity, which leads to an eroded appearance and the generation of fissures on the surface of the solid. The impregnations of the solid with hydrogen peroxide and sodium hypochlorite solutions (Figure 1c,d, respectively) determine a partial pulverization of the activated carbon grains, as fine particles on the surface of the bigger activated carbon grains can be observed. According to the micrographs, the oxidation with sodium hypochlorite is apparently the most severe treatment, in terms of the change in the structural aspect.

### 2.2. Physicochemical Characteristics of Activated Carbon

**Figure 2:**
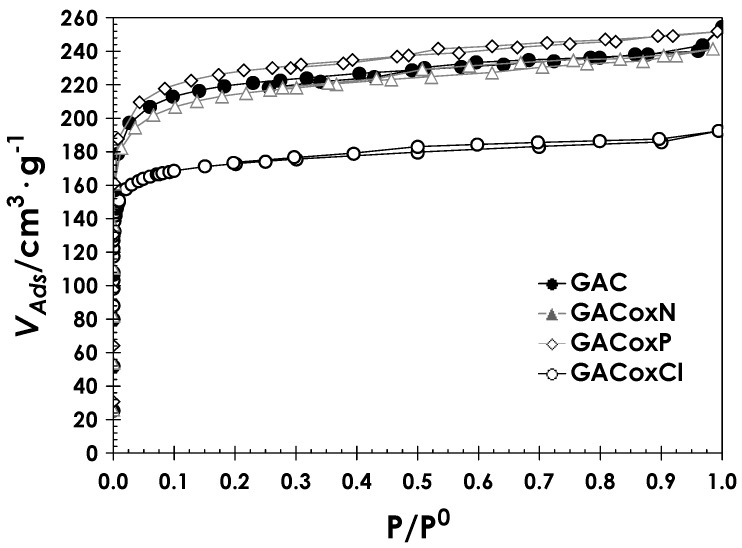
N_2_ adsorption–desorption isotherms at −196 °C, for all the activated carbon set.

The N_2_ adsorption–desorption isotherms of the activated carbon set are presented in Figure 2. The isotherms are essentially of type I(a), according to the International Union of Pure and Applied Chemistry (IUPAC) classification, characteristic of microporous solids. The isotherms are concave with respect to the relative pressure axis and the micropores volume limited the N_2_ amount adsorbed; the adsorption potential in the micropores is high, favored by its size and the establishment of adsorbate–adsorbent favorable interactions [42]. In microporous solids, micropore filling is carried out at relative pressures below 0.1, but at this pressure, it is difficult to discriminate monolayer formation processes from micropore filling. Additionally, within the GAC series, GACoxN and GACoxP solids have a relatively more open elbow at low relative pressures (P/P^0^ < 0.1), which indicates wide and different pore size distribution with respect to the starting GAC.

All the activated carbon textural parameters were evaluated from the N_2_ adsorption isotherms experimental data by application of Brunauer-Emmett-Teller (*BET*), Dubinin-Astakhov (*DA*), and density functional theory (*DFT*) models. In particular, for the determination of the pore size distributions (PSD) of the adsorbents, two different microscopic models (i.e., NLDFT and QSDFT) were used, which allow describing the adsorption and the behavior of fluids in the solid pores at molecular level. The main difference between the NLDFT and QSDFT models is the type of adsorbate–adsorbent interaction, based on the energetic and geometric characteristics of the surface. In detail, NLDFT model assumes flat, structureless, graphitic pore walls, while the QSDFT method takes into account the effects of surface roughness and heterogeneity. The differences in fitting errors of NLDFT and QSDFT model application can be considered as a reliable way to determine the applicability of a model to adequately describe an activated carbon having an unknown morphology. Table 1 presents the fitting error for different model application, for each different hypothesis on pore geometry; the data were calculated by AsiQwin software (Quantachrome Instruments, Boynton Beach, FL, USA) [42,43].

**Figure 3:**
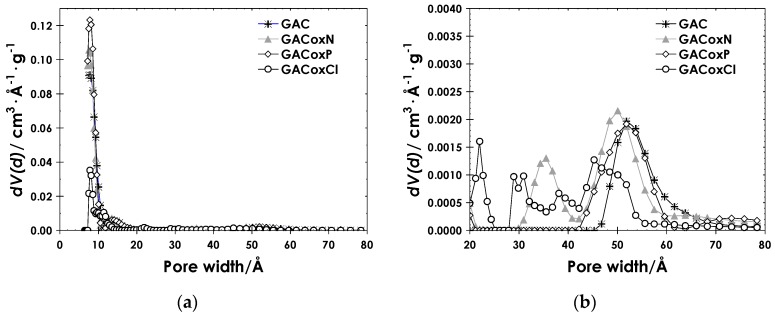
Pore size distribution of (**a**) GAC, GACoxN, GACoxP, and GACoxCl; (**b**) Zooming of a graphic in the range 20–80 Å.

For all the activated carbon samples and each pore geometry, the QSDFT model presents better fits to the experimental data than the NLDFT model, being the fitting error always lower. This result suggests that all the activated carbons have a marked heterogeneous surface, characterized by high roughness and different geometry of pores. Indeed, a better fit of the experimental data to the QSDFT model and the kernel describing a combined pore system (slit and cylindrical) with an average error rate between 0.039% and 1.033% versus 0.0062–2.425% calculated for the NLDFT model and the same kernel, can be observed. In conclusion, the QSDFT model presents significant advantages with respect to NLDFT for the description of the experimental N_2_ isotherm and for the determination of the PSD of the activated carbons investigated, as they proved to be geometrically and chemically heterogeneous (cf. SEM analyses). Hence, it was adopted to retrieve the PSD of the activated carbons, as reported in Figure 3.

For all the activated carbons, the pore dimensions range between 7 and 80 Å (i.e., 0.7 to 8 nm), and the highest contribution is by far in the micropore region (i.e., d < 25 Å). A secondary smaller contribution of larger pores between 30 and 70 Å can be observed in all the samples, which is retained upon all the surface modification of GAC but appears to be shifted also towards mesopores for GACoxN and towards micropores for GACoxCl. The effect of the oxidizing agents is mainly evident in the volume of micropores with dimensions of approximately 10 Å, which remains almost constant for all the samples, except for a slight increase in the case of GACoxP. However, for the latter sample, a significant decrease in the volume of wider micropores is also evident.

The attack of the oxidizing agents is carried out mainly on carbon atoms located in the opening of the pores or on the outer surface of the solid, because for these atoms, the cohesive forces are not compensated, as in the atoms located in the inner surface of the solid. The agents used for the modification of surface chemistry mainly act by including oxygen atoms on the surface of the activated carbon. The inclusion of oxygenated surface groups can produce a decrease in the number of pores, which the nitrogen can access, hence determining a worsening of the textural parameters. Moreover, a significant effect on the surface chemistry is also determined because of oxygenated group insertion.

The textural properties of the activated carbons are presented in Table 2, while in Table 3, the result of Boehm titration and pH_PZC_ determination are reported.

In general, the BET area values ranged between 687 and 871 m^2^·g^−1^, while micropore volumes were between 0.26 and 0.36 cm^3^·g^−1^. The differences in the textural characteristics evidence the effect on the textural properties of the activated carbons of the oxidizing agents used, in function of the oxidizing force of the modifying agent used (either HNO_3_, H_2_O_2_, or NaOCl), as discussed in the following. The solid treated with nitric acid (GACoxN) exhibits a slight decrease in BET surface area, likely due the formation of surface oxygen groups located at the edges of the pore openings, which limits the accessibility of the nitrogen molecule to the porous structures [44,45,46]. During the process of surface modification by oxidation in aqueous solution, several phenomena are involved, such as the formation of surface groups, and the opening of new porous structures along with the widening and deepening of the existing structures. The equilibrium between these processes finally determines the effect on the apparent surface area of the solids [42,45]. According to Boehm titration, the surface groups developed on GACoxN are mainly carboxylic acid (0.197 molecules nm^−2^), as confirmed in the literature [46,47]. Coherently, the pH_PZC_ is significantly lower. The modification exerted by HNO_3_ also included the collapse of some porous structures, the latter effect explaining the increase in the volume of mesoporosity observed in the histogram of Figure 3b. Parallel to the increase in the acidic groups, a decrease in the basic character of the surface is also observed, as a product of the neutralization of basic groups by the nitric acid dissolved in the treatment solution [46].

On the other hand, the apparent *BET* surface area of GACoxP increased with respect to the parent GAC. According to the distribution of pores after the oxidation process (Figure 3c), an increase in the volume of pores with dimensions close to 1 nm occurred as a result of the oxidation process. Hence, this treatment led not only to the formation of oxygenated surface groups (also in this case represented by the carboxylic) but also to the opening of new porous structures [44], which may be related to the rupture of the carbon grains due to the reaction between hydrogen peroxide and activated carbon. Furthermore, the GACoxP sample has also the highest micropore volume, and its pores are less energetic than those of the other two oxidized solids. Overall, the treatment with hydrogen peroxide determines a slighter increase in the acidity of the activated carbon, also producing a little increase in the total basic groups. Consequently, the pH_PZC_ resulted slightly higher with respect to the GAC sample.

Finally, the solid subjected to the oxidation treatment with sodium hypochlorite solution (GACoxCl) presents a greater decrease in both *BET* apparent surface area and micropore volume with values of 687 m^2^·g^−1^ and 0.27 cm^3^·g^−1^, respectively, corresponding to a decrease between 18% and 21% with respect to GAC. According to the Boehm titration data, the NaOCl treatment increased the acidity parameter by promoting the formation of functional groups, like phenolic groups, which present an increase of approximately seven times the initial concentration of the original solid (Table 3). A pH_PZC_ higher than the other oxidized samples is related to the acid strength of the generated groups, in this case phenolic groups, which are weaker than carboxylic acid or lactone groups [47].

### 2.3. Cd (II) Adsorption Tests

#### 2.3.1. Adsorption Isotherms

Adsorption equilibrium correlates the amount adsorbed per unit weight of adsorbent and equilibrium concentration of adsorbate, which provides important information to characterize the adsorption system. Indeed, adsorption isotherms define the limits of the performances of an adsorbent solid and have an influence in its kinetic too, as will be discussed in the next section.

**Figure 4:**
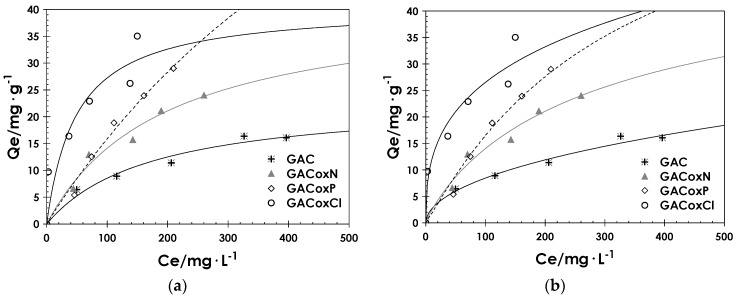
Cadmium adsorption isotherms from aqueous solution on GAC, GACoxN, GACoxP, and GACoxCl, T = 25 °C and pH = 6. The lines correspond to (**a**) Langmuir and (**b**) Sips model.

In Figure 4, cadmium adsorption isotherms are reported for all the set of activated carbons.

The experimental data reports significant differences in the performances of the activated carbon samples, suggesting that all the oxidation treatments have a positive effect on Cd adsorption capacity, which becomes more marked as the equilibrium concentration increases.

In fact, the removal of Cd ions from aqueous solution may be attributed mainly to specific interactions with oxygen-containing functional groups, through metal complex formation on the surface of the carbon, donor-acceptor electron interactions, or both [48]. GACoxCl showed the highest Cd adsorption capacity, likely due to the highest concentration of acidic functional groups on its surface. Specifically, it seems that phenolic groups have a major effect, also explaining the slightly higher adsorption capacity of the GACoxP sample with respect to GACoxN. Another interesting result can be observed from an overall analysis of experimental data: Cd adsorption capacity seems to be not related to the textural parameters, such as BET surface area and micropore volume, as already observed in a previous work dealing with the adsorption of same heavy metal [49]. In fact, the activated carbon with highest Cd adsorption capacity (i.e., GACoxCl) is also the one showing the lowest value of both BET surface area and micropore volume, and the observed ranking between all the adsorbents does not reflect the trend of textural properties.

Different models have been proposed in the literature to describe adsorption phenomena by the fitting of experimental isotherm data, so as to calculate fundamental parameters such as maximum adsorption capacity, favorability, and energetic and affinity parameters. Currently, it should be noted that there is still no model that can fully explain the adsorption and all its properties. However, the availability of a reliable model, which can describe the adsorption capacity over a range of equilibrium concentration of interest for practical application, is an invaluable tool, also for the design of adsorption devices. In this work, the Cd equilibrium data were adjusted to different adsorption models, among those commonly proposed in the pertinent literature. Among them, the best results were obtained for the Langmuir and Sips models, whose model expressions are briefly resumed in the following. 

Langmuir Isotherm model is reported in Equation (1):

(1) $$Q_{e}=Q_{0}\times \frac{K_{L}C_{e}}{1+K_{L}C_{e}}$$

where *Qe* is the adsorbed amount per unit mass of adsorbent (mg·g^−1^), *Ce* is the concentration in the solution at equilibrium (mg·L^−1^), *Q*_0_ is the maximum adsorption capacity, and *K_L_* is the adsorption constant, related to the free energy of adsorption (L·mg^−1^). This isotherm is based on three assumptions: adsorption is limited to monolayer coverage, all active sites are the same, and the ability of a molecule to be adsorbed at a given site is independent of its occupation of neighboring sites [28,30,31,32,33,34].

The equilibrium parameter, *R_L_*, describes the nature of the Langmuir isotherm. *R_L_* is a dimensionless constant referred to as separation factor and is defined by the following equation:

(2) $$R_{L}=\frac{1}{1+K_{L}C_{0}}$$

where *C*_0_ (mg·L^−1^) is the initial concentration. If *R_L_* > 1 the isotherm is unfavorable, linear if *R_L_* = 1, and favorable if (0 < *R_L_* < 1) [33].

Sips Isotherm model is reported in Equation (2):

(3) $$Qe=\frac{Q_{s}K_{s}C_{e}^{n_{s}}}{1+K_{s}C_{e}^{n_{s}}}$$

where *Qe* is the adsorbed amount at equilibrium (mg·g^−1^), *Ce* is the concentration in the solution at equilibrium (mg·L^−1^), *Q_S_* the Sips maximum adsorption capacity (mg·g^−1^), *K_S_* the Sips affinity constant (L·mg^−1^), and *n_S_* is the Sips model exponent, which can be interpreted as the heterogeneity factor. A value greater than 1 indicates a heterogeneous system, values close to 1 indicate a material with relatively homogenous binding sites, and, in this case, the Sips isotherm reduces to the Langmuir equation. In addition, when either *Ce* or *K_S_* approaches 0, this isotherm reduces to the Freundlich isotherm. This isotherm is also known as Freundlich–Langmuir isotherm equation; it correctly addresses one of the incongruence of the Freundlich model (i.e., the infinite increase of the adsorbed amount with the increase of the concentration), by including a finite limit when the concentration is sufficiently high [28,30,31,32,33,34].

In Figure 4, the good fitting of both the Langmuir and Sips models is shown. It is worth observing that, even if in the range of investigated concentration, the sample GACoxCl has the highest Cd adsorption capacity, data extrapolation by Langmuir model indicates the GACoxP as the best performing. However, this result should be experimentally confirmed.

The fitting parameters of the models were calculated by the Rosenbrock quasi-Newton optimization method included in the STATISTICA^®^ software. Table 4 summarizes the parameters resulting from the fitting of the adsorption data by applying the models.

As can be observed, the values of the coefficient of determination are comparable for both the models and for all the tested activated carbons. However, the Langmuir model allows the calculation of a lower number of parameters.

The corresponding *R_L_* values ranged between 0 and 1 for all the isotherm data, indicating that adsorption process was favorable [31].

The Sips model incorporates three parameters and, in addition, combines elements of the Langmuir and Freundlich equations, adsorption mechanism being a hybrid, which does not follow the ideal monolayer adsorption. The *n_S_* parameter deviates from 1, which denotes the heterogeneity of the surface of the adsorbents [32].

#### 2.3.2. Fixed-Bed Column Studies

The efficiency of an adsorption process mainly depends on the thermodynamic aspects of solute-solvent-sorbent interactions and on transport phenomena, involving a diffusive-convective transport within the liquid bulk and porous media. Once the textural and thermodynamic properties of the adsorbents were assessed, specific dynamic tests were carried out in a fixed-bed column in order to define their performances in real operating conditions. Indeed, the dynamic behavior of a specific adsorbent is significantly influenced by its porous structure and equilibrium adsorption capacity toward the target compound. Hence, similarly to what made for the equilibrium tests (Figure 4), the dynamic behavior of the adsorbents was investigated at constant operating conditions, so as to allow for a thorough comparison. These conditions were selected through both literature/case study surveys and preliminary tests. In detail, the Cd initial concentration was set at 50 mg·L^−1^, which can be considered as a typical composition of Cd-contaminated water. The liquid flow rate (*Q*) was set at 1.8 L h^−1^, corresponding to a superficial velocity of about 6.4 mm·s^−1^, which is slightly higher than the corresponding values adopted for fixed-bed experiments in solid–liquid applications [34,35,37,50]. This choice allowed operating in a regime of fast external mass transfer, thus allowing the exploitation of intra-particle mass transfer rate (vide infra).

Concerning the adsorbent, constant bed height (40 cm) and particle size (500 µm) were adopted. In particular, the particle size of the adsorbent is a crucial parameter that can affect the feasibility of an operation of adsorption, e.g., by influencing the mass transfer rates. In a previous theoretical work [51], it was demonstrated that, in similar working conditions, a particle diameter of 300 µm allows a significant decrease of the internal mass transfer resistance on the overall adsorption kinetics. In order to quantify this effect, preliminary dynamic tests (not reported) showed that the width of mass transfer zone (*MTZ*) of the fixed-bed significantly decreased when the mean particle diameter decreased from 1 mm to 0.1 mm. However, a correct choice should take into account the effect on the pressure drops, which are obviously higher for smaller particle diameters. Moreover, a proper dc-to-dp ratio (≥15) should be adopted, in order to prevent any by-pass phenomena on column walls [52]. Hence, a compromise value (500 µm) was adopted in all the experimental runs.

**Figure 5:**
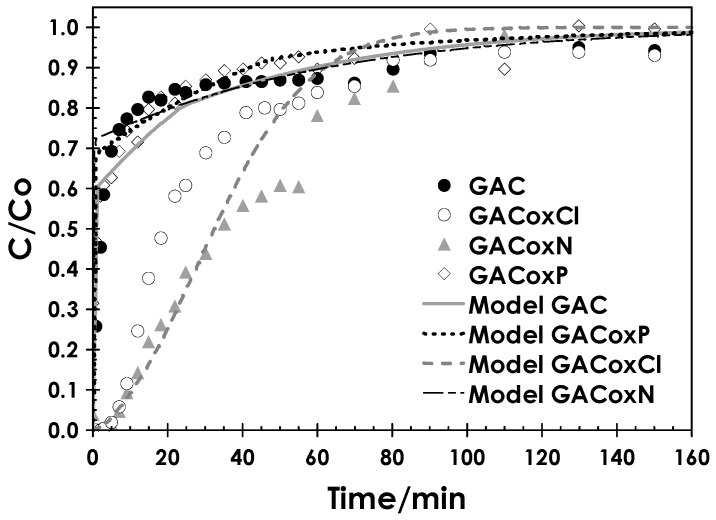
Cd dynamic adsorption tests onto GAC, GACoxCl, GACoxN, and GACoxP adsorbents (dp = 500 µm). *T* = 25 °C, *C*_0_ = 50 mg·L^−1^; *Q* = 1.8 L·h^−1^.

In Figure 5, the dynamic adsorption tests carried out on the different adsorbents investigated are reported.

Experimental results show two different behaviors for the adsorbents. The GAC and GACoxP samples present far lower breakpoint time, as the outlet concentration rises up in the very first working time. This means that, in the investigated conditions, the *MTZ* is wider than bed height, leading to a greater-than-zero Cd concentration in the effluent in the first instant of the dynamic test. On the other hand, GACoxCl and GACoxN show a significantly higher value of the breakpoint time. This result is likely due to the difference in adsorption capacity, which is the highest for the GACoxCl sample. The result of the experimental test on GACoxN seems to be ascribable to a different effect; its equilibrium adsorption capacity at 50 mg·L^−1^ is almost the same as for the GAC and GACoxP samples, hence a similar trend was expected. In fact, for the GACoxN sample, the experimental pH in the fixed-bed test (i.e., pH = 6.8) resulted higher than the corresponding value observed during the thermodynamic test (i.e., pH = 4.2). As demonstrated in Erto et al. [49], equilibrium pH exerts a significant effect on Cd adsorption capacity, due to the possible competition effects arising with the hydronium ions. Hence, it is probable that the unexpected experimental trend shown by GACoxN in the fixed-bed test is due to a higher adsorption capacity during fixed-bed tests determined by the higher operating pH.

Moreover, kinetic adsorption data showed that the slope of the sigmoid results greater for GACoxCl with respect to GACoxN, thus indicating faster mass transfer phenomena for this sample. However, the textural properties of the samples do not appear to be significantly different, hence it can be hypothesized that this effect is also related to a higher adsorption capacity (i.e., higher driving force). Conversely, the GAC and GACoxP samples showed a very similar behavior, but the great extent of the *MTZ* hardly allows the retrieval of any significant indications about the kinetic parameters. For this reason, and in order to give further insights on the kinetic parameters, a modelling analysis of the experimental data set was made.

The dynamic model used to interpret the experimental breakthrough curves included external film diffusion and intraparticle mass transport, while the stage of intrinsic adsorption pseudo-reaction (kinetic) on the active sites of adsorbent surface is neglected, as it is assumed to be instantaneous [53].

The hypotheses adopted for model construction are reported in the following [51,53].

- A plug-flow pattern with axial dispersion (and negligible radial dispersion);
- Isothermal process due to the relatively high heat capacity of water;
- The adsorbent particles have an isotropic spherical shape;
- The accumulation of pollutant in the liquid contained in the pores of the adsorbent material is negligible
- A constant axial velocity.

Under the cited hypotheses, the mass balance equation for the adsorbing system considered can be written as:

(4) $$\varepsilon_{b}\frac{\partial c}{\partial t}+\rho_{b}\frac{\partial \omega }{\partial t}=-u\frac{\partial c}{\partial x}+\varepsilon_{b}D_{ax}\frac{\partial^{2}c}{\partial x^{2}}$$

In which *c* and *ω* are the concentrations of the contaminant in the fluid flow and on the solid, *ρ_b_* is the bed bulk density, u is the axial superficial velocity, *ε_b_* is the fixed-bed porosity, and *D_ax_* the axial dispersion coefficient.

The resolution of the mass balance equation requires estimating the diffusional mass transfer and an experimental relationship that correlates the adsorption capacity of the pollutant with its concentration in the fluid phase (i.e., an adsorption isotherm). In this framework, the Langmuir model was adopted, as it provided a very good description of thermodynamic data (cf. Section 2.3.1).

The mass transfer equation describing the overall adsorption on the solid is:

(5) $$\rho_{b}\frac{\partial \omega }{\partial t}=MTC_{tot}\left(c-c^{*}\right)$$

where all the contributions to mass transfer are grouped into a global coefficient *MTC_tot_* and c^*^ (*ω*) is the concentration of the liquid in equilibrium with the loading *ω*.

In order to study the adsorption dynamics on porous solid, it is possible to split the overall mass transfer from liquid to solid in various steps, namely external film transport, pore volume diffusion, surface adsorption, and surface diffusion. A thorough individuation of the influence of each step is of great importance for a correct description of the adsorption kinetics and for design purposes. As an example, the presence of a non-negligible surface diffusion can have a significant effect on the overall rate of adsorption, increasing it up to 20 times or more [54].

The external mass transfer coefficient per unit volume of bed *MTC_ext_* can be expressed as [36].

(6) $$MTC_{ext}=\frac{ShD}{d_{p}}\frac{6\left(1-\varepsilon_{b}\right)}{d_{p}}$$

In which D is the molecular diffusivity, and *Sh* is the Sherwood number, calculable through empirical relationships available in the literature [51,55].

For the internal mass transfer coefficient $k_{n}^{c}$ both pore diffusion and surface diffusion were considered, occurring in parallel (in turn in series with the external transport):

(7) $$k_{n}^{c}=\frac{15\Psi_{p}\left(1-\varepsilon_{b}\right)\varepsilon_{p}D_{p}}{\Lambda \cdot r_{p}^{2}}+\frac{15\Psi_{s}D_{s}}{r_{p}^{2}}$$

where $\varepsilon_{p}$ and $r_{p}$ are the particle porosity and radius, $D_{p}$ and $D_{s}$ are the pore and surface diffusivities, $\Lambda =\rho_{b}\omega^{*}/c_{0}$ is the partition ratio (in which $\omega^{*}\left(c_{0}\right)$ is the loading in equilibrium with the inlet analyte concentration $c_{0}$), $\Psi_{p}$ and Ψ are two parameters of pore and surface diffusion respectively, which can be expressed as a function of the separation factor $R=1/\left(1+K_{L}c_{0}\right)$ and partition ratio [55].

$D_{p}$ was calculated via empirical relationships which take into account the hydrodynamic resistance and the steric interactions with the walls of adsorbent pores, as reported in Vaccinate et al. (2014) [51]. The surface diffusivity $D_{s}$ as estimated through a best-fitting analysis of experimental data; both these parameters were considered as constant with surface coverage.

Finally, the global mass transfer coefficient can be calculated as follows [32,36]:

(8) $$MTC_{tot}={\left(\frac{1}{MTC_{ext}}+\frac{1}{\Lambda \cdot k_{n}^{c}}\right)}^{-1}$$

The model was numerically solved adopting the finite differences method and a one-dimensional approach, which is appropriate when the length–diameter ratio of the column is sufficiently high. In particular, the 1st order Upwind Differencing Scheme was adopted for the first-order derivatives and the 2nd order Central Difference Scheme for the second-order ones (dispersive terms).

A comparison between experiments and model results is reported in Figure 5, while in Table 5 the kinetic fitting parameters are listed for each adsorbent.

As can be observed, different fitting results were obtained for the single adsorbents. The best fitting results were obtained for the GAC and GACoxP adsorbent, for which a very good approximation of experimental data can be retrieved from Figure 5. For GACoxCl, the model does not describe accurately all the breakthrough curve, as it underestimates mass transfer in the first part of the breakthrough and, conversely, overestimates it in the final part. Indeed, the surface diffusion obtained by model fitting can be considered as an average value, which assumes increasing values along with the adsorbent coverage, as described in the literature [51,55]. Finally, the modelling analysis for GACoxN was evidently affected by the already highlighted difference between the pH values recorded during thermodynamic and kinetic tests. For this reason, a theoretic model curve was plotted assuming a conservative value of the superficial diffusion, just to describe the curve and allow for comparisons with the other adsorbents. An overall analysis of modelling data suggests that, in the adopted experimental conditions, mass transfer is controlled by internal diffusion, being pore diffusion (variable in a narrow range for all the adsorbents, 1.17 × 10^−10^–1.19 × 10^−10^ m^2^·s^−1^) and surface diffusion acting in parallel. Moreover, the retrieved values of the surface diffusion are in the range of typical application for heavy metal adsorption [54]. 

Finally, a joint evaluation of both thermodynamic and kinetic results allowed individuating the GACoxCl sample as the best-performing, because it has a sensibly higher adsorption capacity coupled with the highest global mass transfer coefficient.

## 3. Materials and Methods

### 3.1. Activated Carbon

Activated carbon (GAC) was obtained from coconut shells and CO_2_ physical activation. The solid was sieved to a particle size of 1 mm and washed with a dilute solution of 0.01 M HCl, to remove impurities and part of the ashes. After that, the sample was washed with distilled water to remove the excess of acid, dried for 24 h at 90 °C and stored in plastic containers under nitrogen flow. Subsequently, the GAC was subjected to three alternative oxidation processes by impregnation with aqueous solution of different oxidants:

Nitric acid, HNO_3_, oxidation: The previously washed GAC was mixed with a 6 M solution at 1:2 impregnation ratio, then the mixture was heated at its boiling temperature. The sample was named as GACoxN.

Hydrogen Peroxide, H_2_O_2_, oxidation: GAC was mixed with 10 M hydrogen peroxide solution at 1:2 impregnation ratio room temperature. The sample was named as GACoxP.

Sodium Hypochlorite, NaClO, oxidation: GAC was mixed with 2 M sodium hypochlorite solution at room temperature. The sample was named as GACoxCl.

After treatment, all the samples were washed with distilled water until the pH of the water resulted constant.

### 3.2. Adsorbent Characterization

#### 3.2.1. Textural Properties

Activated carbon textural parameters, such as BET surface area, pore volume, and pore width, were evaluated by physical adsorption of N_2_ at −196 °C and CO_2_ at 0 °C in an automatic Autosorb 3B apparatus (Quantachrome Instruments, Boynton Beach, FL, USA). Starting from raw data, Brunauer-Emmett-Teller (BET), Non-Local Density Functional Theory (NLDFT), and Quenched Solid Density Functional Theory (QSDFT) models were applied and compared for BET surface area and pore geometric properties determination.

#### 3.2.2. SEM Analysis

SEM micrographs were obtained on a JEOL Model 6490-LV Scanning electron microscope (JEOL, Akishima, Tokyo, Japan). The procedure consists of placing small fragments of the sample on a metallic surface to obtain the maximum contrast in the photograph. The sample is transferred to the SEM chamber, and an acceleration voltage of 5 kV is observed at different magnification (between 100 and 10,000×).

#### 3.2.3. pH at the Point of Zero Charge (pH_PZC_)

The pH value at the point of zero charge, pH_PZC_, was determined by the mass titration method. Different weighed amounts of activated carbon (0.010–0.600 g) were placed in a series of 50-mL glass bottles and 10 mL of 0.1 M NaCl solution was added to each of them. The bottles were stoppered and left under constant stirring for 48 h, and the pH value of each solution was measured using a CG 840B Schott pH meter (Xylem Analytics Germany Sales GmbH & Co. KG, Weilheim, Germany) [39].

#### 3.2.4. Boehm Titration Method

Activated carbon surface chemistry was characterized by Boehm titration analysis, in order to quantify the content of both acidic and basic surface functional groups. For this purpose, 0.500 g of each activated carbon was put in contact with 50 mL of either 0.1 M NaOH, Na_2_CO_3_, or NaHCO_3_ solution to determine the different acidic groups. Similarly, 50 mL of 0.1 M HCl was added to determine the total basicity. The mixtures were kept at a constant temperature of 25 °C with constant stirring for 48 h. Subsequently, a 10-mL aliquot of each working solution was titrated with 0.1 M standard solutions of HCl or NaOH for basic and acidic group determination, respectively. The titration curves were obtained in a CG 840B Schott pH meter.

### 3.3. Cd (II) Adsorption Tests

For all the tests, Cd (II) solutions were prepared starting from CdSO_4_·3H_2_O (Sigma Aldrich, St. Louis, MO, USA, analytical grade reagent) in double distilled water. The Cd (II) concentration investigated in this study ranged between 50 and 500 mg·L^−1^. Cd concentrations were determined in a Perkin Elmer atomic absorption spectrophotometer (Analyst 300) (Perkin Elmer, Waltham, MA, USA).

#### 3.3.1. Adsorption Isotherms

Adsorption samples consisted in 50 mL of Cd (II) solution at different initial concentrations (100–500 mg·L^−1^) put in contact with constant amount of activated carbon (0.500 g). The mixtures were adjusted to pH 6 and maintained at a constant temperature of 25 °C for 100 h until equilibrium was reached, occasionally being stirred. At the end of this equilibration time, the mixture was filtered to remove the activated carbon and the Cd (II) concentration was determined by atomic absorption.

#### 3.3.2. Fixed Bed Column Studies

The experimental setup of the fixed-bed column studies consisted of a storage tank containing the cadmium solution (C = 50 mg·L^−1^), which was continuously fed to a glass column having a 1.0 cm internal diameter (dc) and 40 cm of length, packed with granular activated carbon (particle size, dp: 500 µm). After passing through the column, the Cd (II) concentration of the solution was determined at different intervals of time by atomic absorption; finally, it was discharged into a collection tank. The volumetric flow rate was kept constant at 30 mL·min^−1^ and regulated with a peristaltic pump (Watson Marlow, Wilmington, MA, USA).

## 4. Conclusions

In this work, adsorption studies were carried out on cadmium removal from aqueous solution by activated carbons with different surface chemistry deriving from different oxidation treatments. The differences in the textural characteristics of the obtained adsorbents evidence the effect of the oxidizing agents used on the textural properties of the activated carbons in function of the oxidizing force of the modifying agent used (either HNO_3_, H_2_O_2_, or NaOCl). Similarly, the oxidation treatments determined an increase in the surface functional groups, especially those acidic (maximum for GACoxN), and a decrease in the pH_PZC_ (except for the GACoxCl sample). 

The experimental data reports significant differences in the adsorption performances of the oxidized samples with respect to the parent material, suggesting that all the oxidation treatments have a positive effect on Cd adsorption capacity, which becomes more marked as the equilibrium concentration increases. GACoxCl showed the highest Cd adsorption capacity, likely due to the highest concentration of acidic functional groups on its surface, in particular the phenolics, and despite the lowest BET surface area and micropore volume among all the adsorbents and the model that was applied. Equilibrium data were satisfactory modelled by Langmuir and Sips models, which allowed correlating the different adsorption capacities with the physicochemical properties of the adsorbents (e.g., heterogeneity).

Dedicated dynamic tests were carried out in a fixed-bed column, in order to investigate the kinetic behavior of the synthesized adsorbents. For this reason, the experimental runs were carried out at constant Cd initial concentration and liquid flow rate. The GAC and GACoxP samples showed a far lower breakpoint time, meaning that, in the investigated conditions, the MTZ is wider than bed height, leading to a greater-than-zero Cd concentration in the effluent in the first instant of the dynamic test. On the other hand, GACoxCl and GACoxN showed a significantly higher value of the breakpoint time. This result is likely due to the difference in adsorption capacity, which is highest for the GACoxCl sample.

Finally, the dynamic tests were analyzed in light of a kinetic model accounting for both external film diffusion and intraparticle mass transport, in turn including the contribution of both pore and surface diffusion. Modelling results suggests that, in the adopted experimental conditions, mass transfer is controlled by internal diffusion and the retrieved values of the surface diffusion are in the range of typical application for heavy metal adsorption.

## Figures and Tables

**Table 1:** Fitting error (%) for different pore geometry (slit, cylindrical, and combined) assuming either homogeneous surfaces (NLDFT) or rough/heterogeneous surfaces (QSDFT).

Sample	Slit Pore (%)		Cylindrical Pore (%)		Combined Pore (%)	
	NLDFT	QSDFT	NLDFT	QSDFT	NLDFT	QSDFT
**GAC**	2.34	1.27	1.97	1.46	1.46	1.03
**GACoxN**	2.22	1.61	2.47	1.61	0.970	0.678
**GACoxP**	2.69	1.15	3.42	2.22	1.31	1.22
**GACoxCl**	0.580	0.096	0.337	0.096	0.118	0.039

**Table 2:** Textural properties of activated carbon.

Model	Sample	GAC	GACoxN	GACoxP	GACoxCl
	Variable				
BET	*S_BET_* (m^2^·g^−1^)	849	815	871	687
	C	117	121	113	155
DA (P/P^0^ < 0.1)	*Vmic* (cm^3^·g^−1^)	0.35	0.35	0.36	0.26
	*Eo* (kJ·mol^−1^)	7.64	8.447	7.659	9.30
	n	1.80	1.40	1.80	2.00
	Pore diameter (Å)	14.2	13.4	14.2	13.4
QSDFT (P/P^0^ 10^−5^ − 1)	*V_P_* (cm^3^·g^−1^)	0.34	0.34	0.35	0.27
	Pore width (mode) (Å)	7.85	7.53	7.85	7.85

*Eo*: Characteristic Energy.

**Table 3:** Density of surface functional groups (molecules nm^−2^) determined by Boehm titration and point of zero charge.

	GAC	GACoxN	GACoxP	GACoxCl
Carboxylic	0.052	0.197	0.106	0.035
Lactonic	0.029	0.039	0.024	0.056
Phenolic	0.061	0.054	0.074	0.350
Total Acidity	0.142	0.290	0.204	0.441
Total Basicity	0.065	0.036	0.073	0.106
Total Groups	0.207	0.326	0.277	0.547
pH_PZC_	5.4	3.4	6.2	7.2

**Table 4:** Fitting parameters of Langmuir and Sips models for cadmium adsorption isotherms from aqueous solution on GAC, GACoxN, GACoxP, and GACoxCl, *T* = 25 °C and pH = 6.

Samples	Langmuir Model				Sips Model			
	*Q*_0_ (mg/L)	*K_L_* (L/mg)	*R^2^*	*R_L_* (50–500 mg·L^−1^)	*Q_S_* (mg/L)	*K_S_* (L/mg)	*n_s_*	*R*^2^
GAC	93.3	0.0012	0.991	0.67–0.17	185	0.0044	0.519	0.993
GACoxN	41.6	0.0051	0.999	0.80–0.29	51.5	0.0063	0.887	0.989
GACoxP	131	0.0014	0.993	0.95–0.67	71.1	0.0022	1.067	0.996
GACoxCl	40.6	0.0203	0.993	0.57–0.12	190	0.0275	0.385	0.970

**Table 5:** Values of the mass transfer coefficients and kinetic fitting parameters of dynamic experimental data.

Adsorbent	*MTC_ext_* (s^−1^)	$k_{n}^{c}$ (s^−1^)	*D_s_* (m^2^·s^−1^)	*MTC_tot_* (s^−1^)
GAC	5.69 × 10^−1^	1.35 × 10^−3^	4.6 × 10^−11^	2.17 × 10^−1^
GACoxP	5.69 × 10^−1^	7.72 × 10^−3^	3.2 × 10^−11^	2.14 × 10^−1^
GACoxN	5.69 × 10^−1^	2.43 × 10^−3^	1.0 × 10^−11^	1.01 × 10^−1^
GACoxCl	5.69 × 10^−1^	9.76 × 10^−3^	4.2 × 10^−11^	3.86 × 10^−1^
